# mTOR inhibitor versus mycophenolic acid as the primary immunosuppression regime combined with calcineurin inhibitor for kidney transplant recipients: a meta-analysis

**DOI:** 10.1186/s12882-015-0078-5

**Published:** 2015-07-01

**Authors:** Xishao Xie, Yan Jiang, Xiuxiu Lai, Shilong Xiang, Zhangfei Shou, Jianghua Chen

**Affiliations:** Kidney Disease Center, The First Affiliated Hospital, Medical School of Zhejiang University, Qingchun Rd, Hangzhou, Zhejiang China

**Keywords:** mTOR inhibitor, Mycophenolic acid, Kidney transplantation, Meta-analysis

## Abstract

**Background:**

A number of studies have provided information regarding the risks and benefits of mammalian target of rapamycin inhibitors (mTOR-I) combined with calcineurin inhibitors (CNI) versus mycophenolic acid (MPA).

**Methods:**

Medline, Embase and the Cochrane Central Register of Controlled Trials were searched. Randomized controlled trials comparing mTOR-I to MPA as the primary immunosuppressive regimen in combination with CNI were selected and meta-analyzed.

**Results:**

Eleven randomized controlled trials consisting of 4930 patients in total were included. No significant difference was observed in the risk of biopsy-proven acute rejection and patient death between the two groups. However, an increased risk of graft loss (relative risk (RR) = 1.20) and inferior graft function (creatinine clearance, weighted mean difference (WMD) = −2.41 μmol/L) were demonstrated in mTOR-I-treated patients. Patients treated with mTOR-I had a higher risk of new-onset diabetes mellitus (RR = 1.32), dyslipidemia, proteinuria (RR = 1.79), peripheral edema (RR = 1.34), thrombocytopenia (RR = 1.97) and lymphocoele (RR = 1.80), but a lower risk of cytomegalovirus infection (RR = 0.40), malignancy (RR = 0.64) and leucopenia (RR = 0.43). There was no difference in diarrhea, anemia, urinary tract infection, polyoma virus infection and impaired wound healing when mTOR-I was compared with MPA.

**Conclusions:**

mTOR-I showed no particular superiority to MPA. Notably, mTOR-I had an increased risk of graft loss when combined with CNI, even when combined with a reduced dose of CNI. Therefore, the optimal dosage strategies for mTOR-I and CNI need to be further explored.

**Electronic supplementary material:**

The online version of this article (doi:10.1186/s12882-015-0078-5) contains supplementary material, which is available to authorized users.

## Background

Kidney transplantation is the best treatment available for most patients with end-stage renal disease; therefore optimal immunosuppression regimens are critical for long-term graft and patient survival. The aim of the introduction of every new immunosuppressive drug is to reduce the incidence of acute rejection, minimize adverse effects and improve long-term patient and graft survival [1, 2]. Since mycophenolic acid (MPA) was introduced into solid organ transplantation, it has largely replaced azathioprine (AZA) as the antimetabolite immunosuppressive of choice in kidney transplantation [3–7]. The current preferred immunosuppressive regimen in most transplant centers is based on the combination of mycophenolic acid (mycophenolate mofetil (MMF) or enteric-coated mycophenolate sodium (EC-MPS)) with a calcineurin inhibitor (CNI: tacrolimus or ciclosporin A) and corticosteroid. Recently, mammalian target of rapamycin inhibitors (mTOR-I; sirolimus and everolimus) with novel mechanisms of action have been used in organ transplantation, providing additional options for new strategies that produce potent immunosuppression to prevent acute rejection, while simultaneously reducing the adverse effects associated with CNI therapies [8, 9].

In 2006, Webster et al. published the first systematic review and meta-analysis on mTOR-I use as the primary immunosuppression in kidney transplantation patients [10]. In that meta-analysis, the authors evaluated mTOR-I versus antimetabolites (MMF and AZA) in *de novo* kidney recipients. The data of this review were limited beyond 2 years post-transplantation, thus the long-term effects of mTOR-I are unclear. Furthermore, in recent years, azathioprine has not routinely been used in most transplant centers. Therefore we have evaluated the latest evidence on the efficacy and safety of mTOR-I versus MPA, in combination with CNI, in kidney transplantation.

## Materials and methods

### Search strategy and selection criteria

A systematic literature search was performed from study inception to June 30, 2014 in the following databases: the Cochrane Central Register of Controlled Trials, Medline and Embase, combined with the following MeSH terms: mammalian target of rapamycin inhibitor, mTOR inhibitor, mTOR-I, rapamycin, Rapamune, everolimus, sirolimus, MPA, mycophenolic acid, MMF, mycophenolate mofetil, CellCept (Roche, Basel, Switzerland), EC-MPS, enteric-coated mycophenolate sodium, calcineurin inhibitor, CNI, ciclosporin/cyclosporine, Neoral, Sandimmune, CsA, tacrolimus, Prograf, FK506 and kidney/renal transplantation. The reference lists from the included studies were examined for further potentially relevant references. Reference lists of the identified papers were also searched for additional relevant studies.

Only the randomized controlled trials in which kidney transplant recipients (with no additional organ transplantation, such as pancreas) receiving CNI-based immunosuppression containing mTOR-I (everolimus or sirolimus) were compared with MPA (MMF or EC-MPS) in the immediate post-transplant period were included. There was no restriction on the language of trial report, type of donor, age of recipients, or dosage of immunosuppressive drugs.

All titles, abstracts and, where required, the full text of identified reports were independently screened by X.X. and Y.J. to determine which studies satisfied the inclusion criteria, with disagreement resolved by discussion. Data on demographic information, study design, interventions and outcomes were extracted independently by the same two authors using a predesigned data extraction form before meta-analysis. Considering duplicated reports of the same trial or patient group, the latest complete publication was identified. However, any other reports that including additional outcome data also contributed to the meta-analysis.

### Outcome measures

The primary outcome measures investigated were biopsy-proven acute rejection (BPAR), graft loss (censored for death and including death with a functioning graft) and patient death. The secondary outcomes were graft function (including serum creatinine, creatinine clearance or calculated glomerular filtration rate [GFR]), infection rates (total infections, urinary tract infection (UTI), cytomegalovirus (CMV) and polyma virus), malignancy, and a range of treatment-related adverse reactions (including hematological, gastrointestinal and biochemical indices, surgical, and cosmetic).

### Assessment of risk of bias

The quality of trials was independently assessed by X.X. and Y.J. using the Cochrane risk of bias assessment tool [11]. The checklist assessed risk of bias in sequence generation, allocation concealment, blinding, attrition, reporting and other areas. Disagreement was resolved by discussion. As for the study quality, all reports from the same trial were assessed and the information pooled.

### Statistical analysis

The Review Manager 5.2 program (Cochrane Collaboration, London, UK) and Stata version 11 (Stata Corp., College Station, TX, USA) were used for meta-analysis. A *P*-value of *P* < 0.05 was considered to be statistically significant. Dichotomous data are expressed as therelative risk (RR), and continuous data are expressed as the weighted mean difference (WMD). All summary effects are presented with 95 % confidence intervals (CI). Analysis used both random and fixed models (depending on the absence or presence of heterogeneity) to estimate the effect size for each outcome measure. If heterogeneity was observed, a random effect model was used. Statistical heterogeneity between trials was assessed using the *I*^2^ and Cochran Q test. An *I*^2^ value greater than 40 % or a Cochran Q *P*-value less than 0.1 indicated significant levels of heterogeneity. Publication bias was assessed by funnel plots and the Egger test.

Subgroup analysis and univariate meta-regression were used where possible to explore the potential sources of heterogeneity based on the specific mTOR-I and MPA used, the induction agents used, the combination of immunosuppressive co-interventions, the different doses of immunosuppressive treatment and the length of follow-up. A *P*-value of *P* < 0.05 was considered statistically significant.

## Results

### Literature search and included trials

**Fig. 1:**
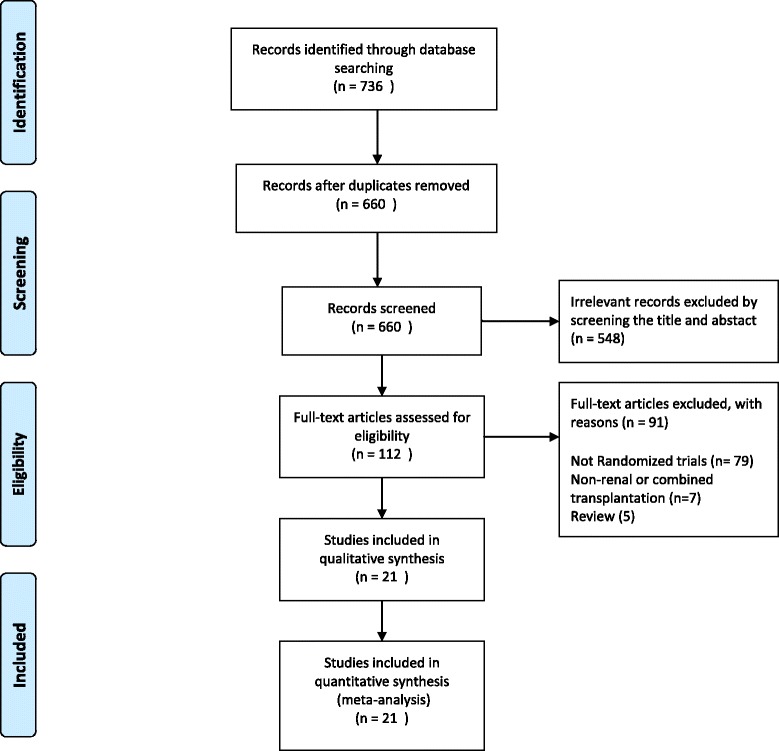
Flowchart showing the process of identification of randomized controlled trials for inclusion in the meta-analysis

The results of the literature search are illustrated in Fig. 1. Twenty-one reports from 11 trials were identified [12–22], with a total of 4930 randomized participants.

All of the included reports were published in English. Seven randomized controlled trials were multicenter trials [12–15, 17, 21, 22]. Seven trials compared sirolimus to MMF [13, 15–20], while 3 trials compared everolimus to MMF [12, 14, 22], and 1 trial compared everolimus to EC-MPS [21]. Five trials had two arms [12, 17, 18, 20, 22], and 5 trials had three arms [13–15, 19, 21]: four of these examined the effects of two different doses of mTOR-I compared with MPA, and the other one compared mTOR-I combined with different CNI to MMF combined with CsA. One trial had four arms that investigated sirolimus versus MMF when combined with different CNI [16]. Two trials used a rapid steroid withdrawal immunosuppression protocol, all recipients received no more than 3 doses of methylprednisolone, then steroid therapy was discontinued [16, 20]. Five trials used basiliximab induction [12, 16, 20–22], while 1 trial used daclizumab induction [19], and 5 trials received no induction therapy [13–15, 17, 18]. The basic characteristics of the included trials are summarized in Table 1.

**Table 1:** Characteristics of the included trials

Trials		Multicentre trial		Induction	mTOR-I group		MPA group		Follow-up (years)
	Country		No.(male)		Maintenance	Dose/target level	Maintenance	Dose/target level	
Vitko et al. 2005	Multiple	Yes	588 (380)	None	EVE + CsA + ST	1.5 mg/d	MMF + CsA + ST	2 g/d	3
						3 mg/d			
Lorber et al. 2005	Multiple	Yes	583 (365)	Bas	EVE + RD-CsA + ST	1.5 mg/d	MMF + CsA + ST	2 g/d	3
						3 mg/d			
Mendez et al. 2005	America	Yes	361 (246)	None	SRL + TAC + ST	2 mg/d	MMF + TAC + ST	2 g/d	1
Vitko et al. 2006	Multiple	Yes	977 (624)	None	SRL + TAC + ST	0.5 mg/d	MMF + TAC + ST	1 g/d	0.5
						2 mg/d			
Kumar et al. 2008	America	No	200 (140)	Bas	SRL + TAC	5-10 ng/ml	MMF + TAC	1-3 u/ml	5
					SRL + CsA	5-10 ng/ml	MMF + CsA	1-3 u/ml	
Sampaio et al. 2008	Brazil	Yes	100 (69)	None	SRL + TAC + ST	2 mg/d	MMF + TAC + ST	2 g/d	1
Gurp et al. 2010	Multiple	Yes	634 (408)	None	SRL + RD-TAC + ST	1 mg/d	MMF + TAC + ST	1 mg/d	0.5
Guerra et al. 2011	America	No	150 (99)	Dac	SRL + TAC + ST	6-10 ng/ml	MMF + TAC + ST	2 g/d	8
					SRL + CsA + ST	6-10 ng/ml			
Chhabra et al. 2012	America	No	82 (50)	Bas	SRL + TAC	7-10 ng/ml	MMF + TAC	2 g/d	8.5
Crbrik et al. 2013	Multiple	Yes	833 (557)	Bas	EVE + RD-CsA ± ST	1.5 mg/d	Ec-MPS + CsA ± ST	1.44 g/d	2
						3 mg/d			
Takahashi 2013	Japan	Yes	122 (83)	Bas	EVE + RD-CsA + ST	3-8 ng/ml	MMF + CsA + ST	2 g/d	1

*Bas* basiliximab; *Dac* daclizumab; *EVE* everolimus; *SRL* sriolimus; *TAC* tacrolimus; *CsA* ciclosporin; *ST* steroid; *MMF* mycophenloate mofetil; *Ec-MPS* enteric-coated mycophenolate sodium

RD-CNI, patients in mTOR-I group received a reduced dose of ciclosporin/tacrolimus compared with the MPA group

### Risk of bias

**Fig. 2:**
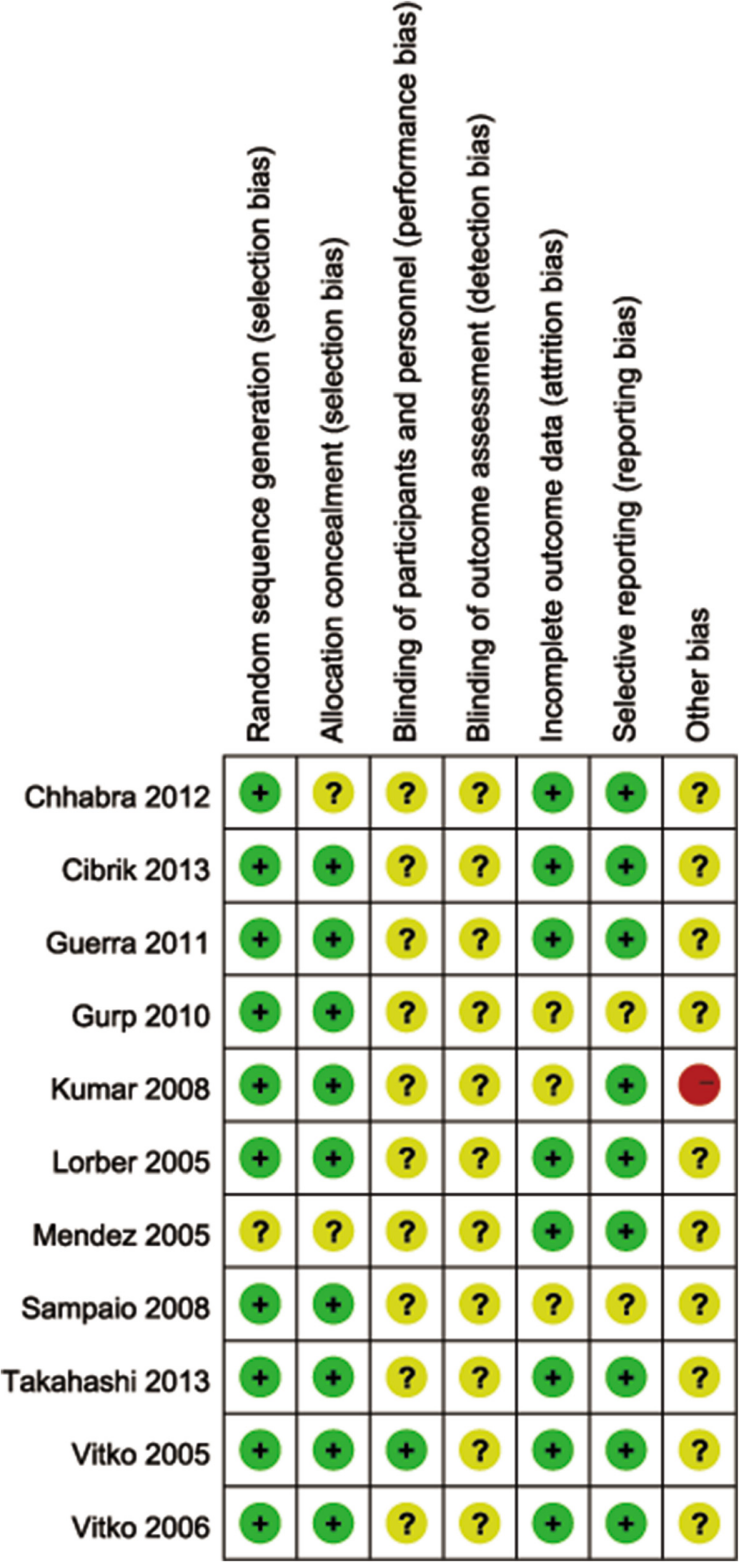
Risk of bias summary: review authors’ judgements about each risk of bias item for each included study

Overall, the quality of the included trials was moderate. The overall assessment of the risk of included trials is displayed in Fig. 2. Ten trials reported adequate sequence generation and for 1 trial this was unclear [13]. Nine trials had low risk of allocation concealment. Only one trial reported blinding of participants and personnel [14]. Eight trials had low risk of bias for reporting incomplete outcome data and for the remaining three trials this was unclear [16–18]. All trials were funded or partially sponsored by a pharmaceutical industry company. Withdraw rates for all studies were < 20 %. All the studies used appropriate statistical tests within their analysis.

### Biopsy-proven acute rejection (BPAR)

**Fig. 3:**
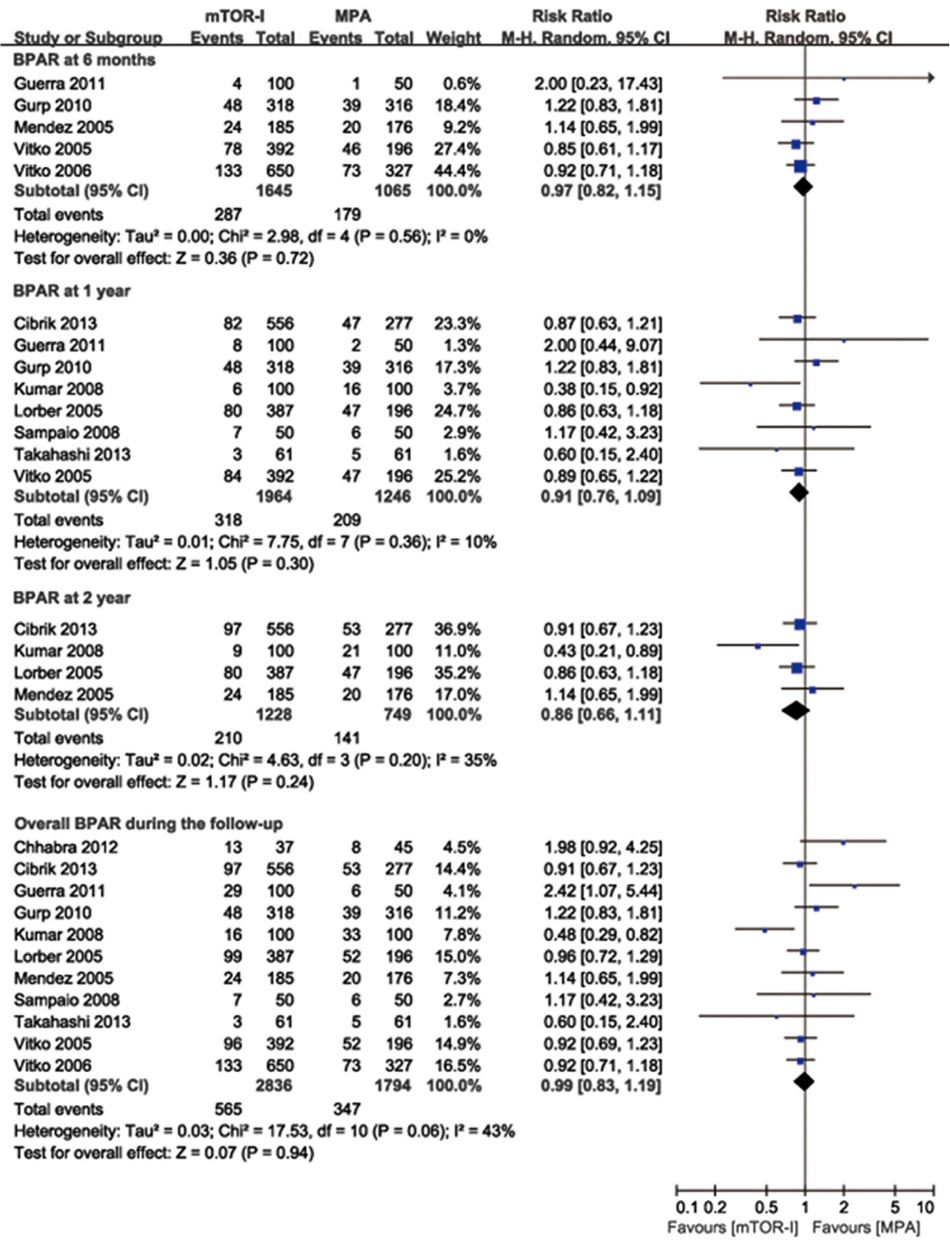
Forest plot showing the relative risk of biopsy-proven acute rejection at 6 months, 1 year, 2 years and at the end of the follow-up period

Eight trials reported the incidence of BPAR [12–16, 19–21], 3 trials reported both clinically defined AR and BPAR [17, 18, 22]. However, only the BPAR was included in the meta-analysis. There was no significant difference in the risk of BPAR when mTOR-I was compared with MPA at 6 months, 1 year, 2 years, and at the end of the follow-up period (6 months: 5 studies, 2710 patients, RR = 0.98, 95 % CI 0.83–1.16, *P* = 0.81; 1 year: 8 studies, 3210 patients, RR = 0.91, 95 % CI 0.78–1.07, *P* = 0.25; 2 years: 4 studies, 1977 patients, RR = 0.86, 95 % CI 0.66–1.11, *P* = 0.24; overall: 11 studies; 4630 patients, RR = 0.99, 95 % CI 0.83–1.19, *P* = 0.94; Fig. 3). Significant heterogeneity was found in the overall risk of BPAR outcome (*P* = 0.06, I^2^ = 43 %) without evidence of publication bias (*P* = 0.39). Subgroup analysis and meta-regression were performed to explore the sources of heterogeneity and to examine whether key trial design features modified the overall BPAR results. The results are summarized in Table 2. There was no evidence that the effects of BPAR differed among subgroups defined according to the key trial design features examined (*P* > 0.05 for all comparisons).

**Table 2:** Meta-regression analysis of potential sources of heterogeneity for the outcome of biopsy-proven acute rejection and graft loss

Covariate	Subgroup		BPAR		Graft loss	
		n.	RR (95 % CI)^a^	*P-*value^b^	RR (95 % CI)^a^	*P-*value^b^
Follow-up	≥3 years	5	1.04 (0.70-1.56)	*P* = 0.96	1.21 (0.99-1.48)	*P* = 0.84
	<3 years	6	0.97 (0.83-1.12)		1.17 (0.90-1.54)	
Mycophenolic acid	MMF	10	1.01 (0.82-1.25)	*P* = 0.75	1.18 (0.99-1.39)	*P* = 0.62
	MPS	1	0.91 (0.67-1.23)		1.36 (0.81-2.28)	
mTOR-I	Sirolimus	7	1.08 (0.78–1.49)	*P* = 0.50	1.14 (0.93-1.41)	*P* = 0.52
	Everolimus	4	0.93 (0.78-1.10)		1.27 (0.99-1.63)	
Induction therapy	None	5	0.99 (0.84-1.16)	*P* = 0.81	1.13 (0.89-1.44)	*P* = 0.54
	Antibody induction	6	1.00 (0.69-1.47)		1.25 (1.01-1.55)	
Calcineurin inhibitor	Tacrolimus	6	1.06 (0.81-1.37)	*P* = 0.12	1.16 (0.90-1.51)	*P* = 0.78
	Ciclosporin	5	0.89 (0.76-1.05)		1.22 (0.98-1.53)	
Steroid withdrawal	Not withdrawn	9	0.99 (0.87-1.12)	*P* = 0.43	1.20 (1.00-1.44)	*P* = 0.96
	Withdrawn rapidly	2	0.95 (0.24-3.77)		1.18 (0.85-1.63)	
CNI dose	RD-CNI in mTOR-I group than MPA	4	0.99 (0.82-1.19)	*P* = 0.93	1.27 (0.95-1.70)	*P* = 0.62
	ED-CNI in mTOR-I group and MPA	7	1.09 (0.93-1.29)		1.16 (0.96-1.40)	

^a^RR < 1 favor mTOR-I

*P-*value^b^ for meta-regression

*RD-CNI* reduced dose of calcineurin inhibitor; *ED-CNI* equal dose of Calcineurin inhibitor

### Graft and patient survival

Patient survival was reported in all trials. There was no significant difference in mortality (11 studies, 4630 patients, RR = 1.17, 95 % CI 0.89–1.53, *P* = 0.27), with no evidence of heterogeneity (*P* = 0.80, I^2^ = 0 %) or publication bias (*P* = 0.97).

**Fig. 4:**
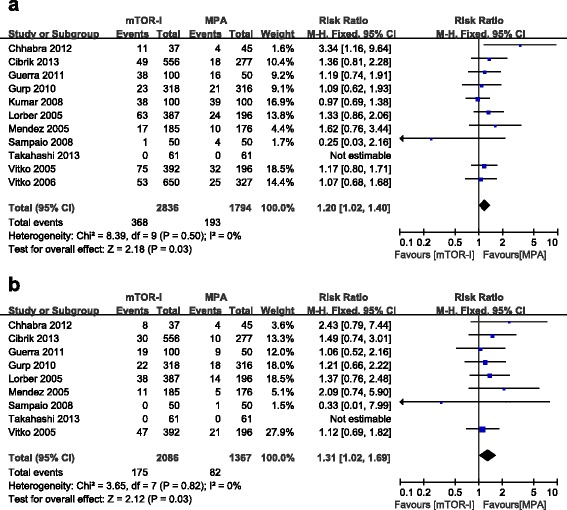
Forest plot showing the relative risk of graft loss (**a**) and death-censored graft loss (**b**)

Overall graft loss (including death with a functioning graft) was reported in all trials. The use of a mTOR-I led to a significantly higher risk of overall graft loss (11 studies, 4630 patients, RR = 1.20, 95 % CI 1.02–1.40, *P* = 0.03) (Fig. 4a). No evident heterogeneity (*P* = 0.50, I^2^ = 0 %) or publication bias (*P* = 0.60) were observed. Death-censored graft loss was reported in 9 trials [12–14, 17–22]. Similarly, patients treated with a mTOR-I showed a significantly increased risk of death-censored graft loss (9 studies, 3453 patients, RR = 1.31, 95 % CI 1.02–1.69, *P* = 0.03) (Fig. 4b). The results of subgroup analysis and meta-regression for overall graft loss are shown in Table 2. No evident difference was observed on the effects of overall graft loss among any subgroups (*P* > 0.05 for all comparisons).

### Graft function

**Fig. 5:**
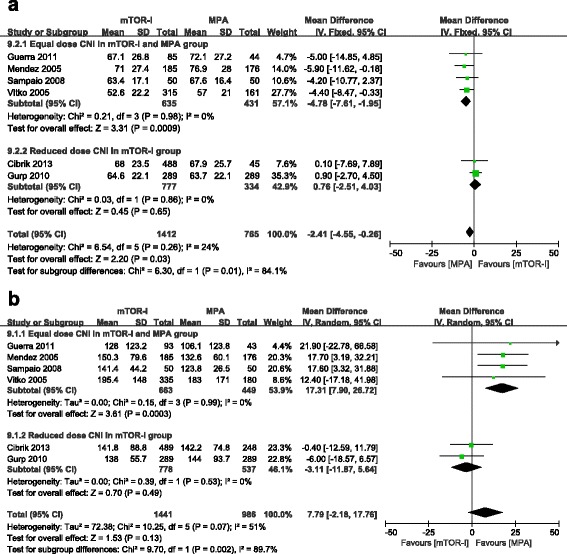
Subgroup analysis for creatinine clearance (**a**) and serum creatinine (**b**), stratified by calcineurin inhibitor dose

Serum creatinine and creatinine clearance (Cockcroft-Gault Formula) was reported in 8 trials [12–15, 17–19, 21]; however, only 6 of them had enough data for meta-analysis (the other 2 trials only described median serum creatinine and a standard deviation could not be calculated). There was no significant difference in serum creatinine between the mTOR-I and MPA groups (6 trials, 2427 patients, WMD = 7.79 μmol/L, 95 % CI −2.18–17.76, *P* = 0.13, I^2^ = 51 %). Patients treated with a mTOR-I demonstrated a lower creatinine clearance (6 trials, 2177 patients, WMD = −2.41 μmol/L, 95 % CI −4.55 to −0.26, *P* = 0.03, I^2^ = 24 %). We performed subgroup analysis for these two outcomes. Serum creatinine was significantly higher and creatinine clearance was significantly lower in the mTOR-I group when combined with equal doses of CNI, as compared with the MPA group (serum creatinine: WMD = 17.31 μmol/L, 95 % CI 7.90–26.72, *P* = 0.0003; creatinine clearance: WMD = −4.78 ml/min, 95 % CI, −7.61 to −1.95, *P* = 0.0009). However, when a mTOR-I was combined with a lower dose of CNI than the MPA group, no significant difference was observed between these two treatment groups (serum creatinine: WMD = −3.11 μmol/L, 95 % CI −11.87–5.64, *P* = 0.49; creatinine clearance: WMD = 0.76 ml/min, 95 % CI −2.51–4.03; *P* = 0.65). No heterogeneity was observed in either subgroup (I^2^ = 0 %) (Fig. 5a and b).

Glomerular filtration rate (GFR) estimated by the Modification of Diet in Renal Disease (MDRD) study equation [23] was also reported in 4 trials. Guerra et al. [19] and Chhabra et al. [20] showed a significantly lower mean GFR in the mTOR-I group compared with the MMF group throughout the entire long-term follow-up period (beyond 8 years). In contrast, Takahashi et al. [22] and Crbrik et al. [21] reported no significant difference in GFR between the two treatments with 1 and 2 years follow-up, respectively.

### New-onset diabetes mellitus (NODM)

New-onset diabetes mellitus (NODM) was reported in 10 trials [13–22]. Patients treated with mTOR-I showed a significantly increased risk of NODM (10 studies, 3550 patients, RR = 1.32, 95 % CI 1.07–1.62, *P* = 0.008). No significant heterogeneity (I^2^ = 4 %) or publication bias (*P* = 0.15) were observed.

Vitko et al. [14] and Cibrik et al. [21] reported that patients treated with a high dose of everolimus (3 mg/d) had a significantly increased risk of NODM when compared with those treated with a low dose of everolimus (1.5 mg/d) or MPA. Similar results were also reported in a Sirolimus study (0.5 mg/d versus 2 mg/d) published by Vitko et al. in 2006 [15].

### Infections

CMV infection was reported in all trials. Patients treated with mTOR-I showed a significantly reduced risk of CMV infection (11 studies, 4622 patients, RR = 0.43, 95 % CI 0.29–0.63, *P* < 0.0001). Heterogeneity was significant (*P* = 0.02, I^2^ = 54 %), but no publication bias was observed (*P* = 0.66). Subgroup analysis and meta-regression also demonstrated that the heterogeneity observed between the studies could not be explained by the type of mTOR-I and MPA used, steroid withdrawal or not, the use of antibody induction or the length of follow-up. No significant difference in the RR of CMV infection was observed for any subgroups examined (*P* > 0.05 for all comparisons).

Urinary tract infection (UTI) was reported in 7 trials [12, 14, 17–21]. There was no significant difference in the incidence of UTI when a mTOR-I was compared with MPA (7 studies, 2962 patients, RR = 1.00, 95 % CI 0.87–1.15, *P* = 0.96), with no significant heterogeneity (*P* = 0.32, I^2^ = 15 %) or publication bias (*P* = 0.94).

Only 2 trials [19, 21] reported the incidence of polyoma virus infection. No significant difference was observed between the two treatment groups (2 studies, 975 patients, RR = 1.05, 95 % CI 0.03–40.89, *P* = 0.98). Heterogeneity was significant and evident (*P* = 0.01, I^2^ = 84 %). Because of the small number of included trials, no subgroup analysis was performed to explore the source of heterogeneity.

### Dyslipidemia

Serum cholesterol and triglyceride levels were also reported in 6 trials [13, 14, 17–19, 21]. Meta-analysis showed that patients treated with mTOR-I had significantly higher cholesterol and triglyceride levels (cholesterol: 6 studies, 1932 patients, WMD = 33.02 mg/dl, 95 % CI 1.11–64.93, *P* = 0.04; triglyceride: 6 studies, 1932 patients, WMD = 36.15 mg/d, 95 % CI 23.65–48.64, *P* < 0.00001). Patients treated with mTOR-I were more likely to require statin therapy (8 studies; 2950 patients; RR = 1.35; 95 % CI 1.18–1.54; *P* < 0.0001). Heterogeneity was evident in all three analyses (Table 3). Subgroup analysis failed to demonstrate a cause for this heterogeneity (data not shown).

**Table 3:** Meta-analysis for secondary outcomes

Outcome	Trials (n)	Patients (n)	Type	RR (95 % CI)^a^	*P*-value	Heterogeneity	
						I^2^ %	*P*-value
New-onset diabetes mellitus	10	3550	Fixed	1.32 (1.07-1.62)	0.008	4	0.40
Urinary tract infection	7	2926	Fixed	1.00 (0.87-1.15)	0.96	15	0.32
CMV infection	11	4622	Random	0.40 (0.27-0.59)	<0.0001	56	0.01
Polyoma infection	2	975	Random	1.05 (0.03-40.89)	0.98	84	0.01
Hyperlipidemia	8	4233	Random	1.72 (1.35-2.20)	<0.0001	60	0.01
Anti-lipid therapy	8	2905	Random	1.35 (1.18-1.54)	<0.0001	72	0.0007
Thrombocytopenia	3	1774	Fixed	1.97 (1.19-3.35)	0.008	37	0.20
Leucopenia	7	3594	Random	0.43 (0.29-0.64)	<0.0001	59	0.02
Anemia	6	2734	Random	1.21 (0.88-1.68)	0.24	81	<0.0001
Proteinuria	7	2861	Fixed	1.79 (1.38-2.31)	<0.0001	0	0.73
Impaired wound healing	6	2374	Fixed	1.55 (0.97-2.47)	0.07	0	0.49
Lymphocele	7	3345	Fixed	1.80 (1.38-2.34)	<0.0001	0	0.71
Diarrhea	7	3729	Random	0.89 (0.64-1.25)	0.50	80	<0.0001
Peripheral edema	5	2752	Random	1.34 (1.08-1.68)	0.009	56	0.06
Malignancy	8	4250	Fixed	0.64 (0.45-0.91)	0.01	32	0.17
				Weighted mean difference			
Serum creatinine (μmol/L)	6	2427	Random	7.79 (−2.18 to 17.76)	0.13	51	0.07
Creatinine Clearance (mL/min)^b^	6	2177	Fixed	−2.41 (−4.55 to −0.26)	0.03	5	0.38
Total cholesterol (mg/dL)	6	1932	Random	33.02 (1.11 to 64.93)	0.04	98	<0.00001
Total triglyceride (mg/dL)	6	1932	Random	36.15 (23.65 to 48.64)	<0.00001	45	0.11

^a^RR < 1 favor mTOR

^b^Cockcroft-Gault Formula

### Hematologic adverse events

Hematologic adverse events, including thrombocytopenia, leucopenia and anemia were reported in several trials. The use of a mTOR-I showed a significantly increased risk of thrombocytopenia (3 studies, 1774 patients, RR = 1.97, 95 % CI 1.19–3.35, *P* = 0.008, I^2^ = 37 %) [13, 14, 21] and a significantly reduced risk of leucopenia (7 studies, 3954 patients, RR = 0.43, 95 % CI 0.29–0.64, *P* < 0.0001, I^2^ = 59 %) [13–15, 18, 20–22]. However, no significant difference in the risk of anemia was observed (6 studies, 2734 patients, RR = 1.21, 95 % CI 0.88–1.68, *P* = 0.21, I^2^ = 81 %) [12, 14, 18, 20–22]. The heterogeneity could not be explained by subgroup analysis.

### Proteinuria

Proteinuria was reported in 7 trials [12, 15–17, 20–22]. Patients treated with a mTOR-I showed a significantly increased risk of proteinuria (7 studies, 2861 patients, RR = 1.79, 95 % CI 1.38–2.31, *P* < 0.0001), with no evident heterogeneity (*P* = 0.73, I^2^ = 0 %).

### Wound related complications

Impaired wound healing (including wound infection and dehiscence) was reported in 6 trials [15–17, 19, 21, 22]. No significant difference was observed when mTOR-I were compared with MPA (6 studies, 2374 patients, RR = 1.55, 95 % CI 0.97–2.47, *P* = 0.07), although patients treated with a mTOR-I had a significantly increased risk of lymphocoele (7 studies, 3345 patients, RR = 1.80, 95 % CI 1.38–2.34, *P* < 0.0001) [12, 14, 15, 17, 19, 21, 22]. Both of these meta-analyses showed low heterogeneity (I^2^ = 0 %).

### Malignancy

Eight trials reported the incidence of malignancy after transplantation [12–16, 18, 20, 21]. The pooled incidence of malignancy was 2.4 % (62 of 2621) in the mTOR-I group and 3.6 % (58 of 1629) in the MPA group. Patients treated with mTOR-I showed a significantly reduced risk of post-transplantation malignancy (8 studies, 4250 patients, RR = 0.64, 95 % CI 0.45–0.91, *P* = 0.01), heterogeneity was not significant (*P* = 0.17; I^2^ = 32 %). Among the 8 trials, 4 trials [12, 15, 16, 18] reported the incidence of post-transplantation lymphoproliferative disease (PTLD). There was no significant difference in the risk of PTLD between the two treatment groups (4 studies, 2394 patients, RR = 0.78, 95 % CI 0.27–2.28, *P* = 0.65), with no significant heterogeneity (*P* = 0.22, I^2^ = 32 %).

### Other adverse events

Diarrhea was reported in 7 trials [12, 15, 16, 18]. No significant difference was observed (7 studies, 3729 patients, RR = 0.89, 95 % CI 0.64–1.25, *P* = 0.50), although heterogeneity was evident (*P* < 0.0001, I^2^ = 80 %). Even if we excluded the trial which used EC-MPS [21], no evident change in the RR (RR = 0.89, 95 % CI 0.58–1.36) was observed and the heterogeneity was still present (I^2^ = 83 %).

Five trials [12, 13, 16, 19, 22] reported the incidence of peripheral edema. Patients treated with mTOR-I showed a significantly higher incidence of peripheral edema (5 studies, 2752 patients, RR = 1.34, 95 % CI 1.08–1.68, *P* = 0.009). Heterogeneity was evident, but not significant (*P* = 0.06, I^2^ = 56 %).

## Discussion

In this meta-analysis, we have combined data from eleven eligible randomized controlled trials involving 4930 patients and examined the efficacy and safety of mTOR-I versus MPA when combined with CNI. Our methodology was robust by searching all possible studies, including non-English language sources, even in the abstract form, and strictly assessing the quality of included trials and by thoroughly investigating sources of potential heterogeneity. Consequently, reliable conclusions can be drawn from the data.

Our meta-analysis demonstrates that the use of mTOR-I or MPA as the primary immunosuppression regimen combined with CNI has no significant effect on the risk of BPAR and patient deaths. However, patients treated with a mTOR-I have an increased risk of graft loss (by 20 %). Creatinine clearance was also reduced by approximately 2.5 ml/min in mTOR-I-treated patients. Subgroup analysis demonstrated that serum creatinine was increased by approximately 17 μmol/L and creatinine clearance was reduced by approximately 5 ml/min in the mTOR-I group when combined with equal doses of CNI as the MPA group, but no significant difference was observed when mTOR-I were combined with a lower dose of CNI than the MPA group. A previous meta-analysis [10] failed to demonstrate a significant difference in the risk of graft loss in kidney transplantation when mTOR-I were compared with antimetabolites (MMF and AZA) (Additional File 1). Furthermore, the data was limited to 2 year after transplantation. We have recognized that the combination of mTOR-I and CNI provide immunological synergy, but that the limitation of this combination in clinical practice is the enhanced nephrotoxicity of CNI. Therefore, the current strategy in clinical practice is to minimize CNI dosage when using a mTOR-I [9]. However, in our subgroup analysis, even when a mTOR-I combined with reduced dose CNI was compared to MPA, no advantage was observed. Notably, there was a relatively higher risk of graft loss (RR = 1.27, *P* = 0.06, Table 2). Some researchers demonstrated that long-term graft survival may be influenced by CNI minimization strategies, although this may increase the incidence of *de novo* donor-specific antibody and antibody-mediated rejection [24, 25]. However, we could not deduce the reason why the graft loss rate is significantly higher in the mTOR-I group.

For secondary outcomes, mTOR-I-treated patients showed an increased risk of NODM (RR = 1.32), especially in the high dose mTOR-I group. This is in agreement with the data from the US renal data system showing that the risk of NODM was significantly higher in all drug combinations that included mTOR-I compared with other therapeutic regimes without this drug [26]. Hyperlipidemia and proteinuria (RR = 1.79) were also more common in mTOR-I-treated patients. Higher cholesterol and triglyceride levels of approximately 33.02 mg/dl and 36.15 mg/dl, respectively, were observed in mTOR-I-treated patients. Evidence from several studies and reviews have demonstrated that dyslipidemia is more common in patients receiving mTOR-I-based regimens, with increased levels of cholesterol and triglycerides, and an increased use of lipid-lowering agents [27–29]. NODM and hyperlipidemia are well recognized risk factors for post-transplant cardiovascular events, all-cause mortality, and graft loss over a period of years after transplantation [27, 30–32]. Most trials included in our analysis did not report the incidence of cardiovascular events, so we are unable to report on this outcome.

Our study also showed that mTOR-I significantly reduced the risk of CMV infections (RR = 0.43), and malignancy (RR = 0.64) compared with MPA. A previous meta-analysis [10] failed to show a difference in the incidence of malignancy between two treatment groups because of the short-term follow-up. Another meta-analysis [33] reported that the CMV incidence under mTOR-I + CNI treatment ranged from 0 % to 10 %, which was significantly lower than with mTOR-I free immunosuppression. Post-transplant malignancy has emerged as a leading cause of morbidity and mortality, especially in patients who have a high or long-term exposure to immunosuppression [34]. There is already both theoretical and experimental evidence in the literature explaining why a mTOR-I might protect against the development of malignancy [35, 36].

When mTOR-I was compared with MPA, the risk of lymphocoele was increased by 76 %, despite a similar risk of impaired healing. A previous review has reported similar results, in that wound healing complications were observed more frequently after mTOR-I became available, particularly in direct comparison with MMF [37]. In our meta-analysis, patients treated with mTOR-I showed a reduced risk of leucopenia (RR = 0.43), but an increased risk of thrombocytopenia (RR = 1.97). Other outcomes, such as diarrhea and anemia, were similar when mTOR-I were compared with MPA.

Our analysis has several limitations. Firstly, the quality of included trials and the lengths of follow-up are variable among trials. Secondly, although the primary outcomes such as BPAR, graft and patient survival were well reported in most trials, many of the adverse outcomes were not reported. The definitions of reported outcomes were often variable case by case or not clearly specified. For example, some trials reported the creatinine clearance (Cockcroft-Gault Formula) to represent graft function, while others reported the calculated GFR (MDRD). Some trials reported the incidence of hypertriglyceridemia and hypercholesterolemia (with variable definitions) or use of lipid-lowering drugs, while some reported the serum levels of triglyceride and cholesterol. As outlined in Table 1, immunosuppression regimens and dose/target level varied among these trials. For example, some trials used a fixed-dose of mTOR-I and MPA, but some used a control-dose based on therapeutic drug monitoring. The use of steroid and the target level of CNI also varied between trials. Thirdly, we have excluded a trial in which 3.2 % of patients received more than one organ transplant (i.e., not kidney alone, so not matching our inclusion criteria) [38]. In this study, Suszynski et al. compared two different doses of sirolimus with MMF with a 10 year follow-up. Although no significant changes in almost all results would have been observed if we included the data from this study in our current meta-analysis (data not shown), we admit that the summary (RR) effects outlined in this article may not represent the true effects of mTOR-I and MPA. Finally, most of the transplant recipients in the trials included in our meta-analysis are older than 18 years of age; therefore, our findings cannot be applied to pediatric patients.

## Conclusions

In summary, according to our meta-analysis, mTOR-I showed no particular superiority compared with MPA, but in fact had an increased risk of graft loss when combined with CNI. Therefore, we suggest that mTOR-I must be used cautiously in *de novo* kidney recipients in combination with CNI, and that the optimal dose strategies of mTOR-I and CNI need to be further investigated.

## Additional file

Additional file 1: Text S1. Included trials and reports. **Figure S1**. Egger’s test for biopsy-proven acute rejection. **Figure S2**. Egger’s test for graft loss. **Figure S3**. Egger’s test for new-onset diabetes mellitus. **Figure S4**. Egger’s test for CMV infection. **Supplemental Table**. Different results between our meta-analysis and the previous study.

## Abbreviations

AZA: Azathioprine
BPAR: Biopsy-Proven Acute Rejection
CMV: Cytomegalovirus
CNI: Calcineurin inhibitor
CsA: Cyclosporine
EC-MPS: Enteric-coated mycophenolate sodium
MPA: Mycophenolic acid
mTOR-I: mammalian target of rapamycin inhibitor
MMF: Mycophenolate mofetil
NODM: New-onset diabetes mellitus
PTLD: Post-transplantation lymphoproliferative disease
RR: Relative risk
UTI: Urinary tract infection
WMD: Weighted mean difference
