# Supplementary material for: mTOR inhibitor versus mycophenolic acid as the primary immunosuppression regime combined with calcineurin inhibitor for kidney transplant recipients: a meta-analysis
Source: BMC Nephrol. 2015 Jul 1;16:91. doi: 10.1186/s12882-015-0078-5 (PMC4486141; doi:10.1186/s12882-015-0078-5)
Supplement: Additional file 1: Text S1. — Included trials and reports. Figure S1. Egger’s test for biopsy-proven acute rejection. Figure S2. Egger’s test for graft loss. Figure S3. Egger’s test for new-onset diabetes mellitus. Figure S4. Egger’s test for CMV infection. Supplemental Table. Different results between our meta-analysis and the previous study. [file 12882_2015_78_MOESM1_ESM.doc]

**Included Trials and Reports:**

**Kumar-2008**

Kumar MSA, Saeed MI, Ranganna K, et al. Comparison of four different immunosuppression protocols without long-term steroid therapy in kidney recipients monitored by surveillance biopsy: Five-year outcomes. *Transplant Immunol* 2008; **20**: 32.

Anil Kumar MS, Heifets M, Fyfe B, et al. Comparison of steroid avoidance in tacrolimus/mycophenolate mofetil and tacrolimus/sirolimus combination in kidney transplantation monitored by surveillance biopsy. *Transplantation* 2005; **80**: 807

**Cibrik-2013(A2309)**

Cibrik D, Silva HT, Jr., Vathsala A, et al. Randomized trial of everolimus-facilitated calcineurin inhibitor minimization over 24 months in renal transplantation. *Transplantation* 2013; **95**: 933.

Tedesco Silva H, Jr., Cibrik D, Johnston T, et al. Everolimus plus reduced-exposure CsA versus mycophenolic acid plus standard-exposure CsA in renal-transplant recipients. *Am J Transplant* 2010; **10**: 1401.

**Chhabra-2012**

Chhabra D, Skaro AI, Leventhal JR, et al. Long-Term Kidney Allograft Function and Survival in Prednisone-Free Regimens: Tacrolimus/Mycophenolate Mofetil versus Tacrolimus/Sirolimus. *Clin J Am Soc Nephrol* 2012; **7**: 504.

Gallon L, Perico N, Dimitrov BD, et al. Long-term renal allograft function on a tacrolimus-based, pred-free maintenance immunosuppression comparing sirolimus vs. MMF. *Am J Transplant* 2006; 6:1617..

**Mendez-2005(PSG)**

Mendez R, Gonwa T, Yang HC, Weinstein S, Jensik S, Steinberg S. A prospective, randomized trial of tacrolimus in combination with sirolimus or mycophenolate mofetil in kidney transplantation: results at 1 year. *Transplantation* 2005; **80**: 303.

Gonwa T, Mendez R, Yang HC, Weinstein S, Jensik S, Steinberg S. Randomized trial of tacrolimus in combination with sirolimus or mycophenolate mofetil in kidney transplantation: results at 6 months. *Transplantation* 2003; **75**: 1213

**Sampaio-2008**

Sampaio EL, Pinheiro-Machado PG, Garcia R, et al. Mycophenolate mofetil vs. sirolimus in kidney transplant recipients receiving tacrolimus-based immunosuppressive regimen. *Clin Transplant* 2008; **22**: 141.

**Gup-2010**

Van Gurp E, Bustamante J, Franco A, et al. Comparable Renal Function at 6 Months with Tacrolimus Combined with Fixed-Dose Sirolimus or MMF: Results of a Randomized Multicenter Trial in Renal Transplantation. *J Transplant* 2010; **2010**.

**Guerra-2011**

Guerra G, Ciancio G, Gaynor JJ, et al. Randomized Trial of Immunosuppressive Regimens in Renal Transplantation. *J Am Soc Nephrol* 2011; **22**: 1758.

Ciancio G, Burke GW, Gaynor JJ, et al. A randomized long-term trial of tacrolimus/sirolimus versus tacrolimums/mycophenolate versus cyclosporine/sirolimus in renal transplantation: three-year analysis. *Transplantation* 2006; **81**: 845.

Ciancio G, Burke GW, Gaynor JJ, et al. A randomized long-term trial of tacrolimus/sirolimus versus tacrolimus/mycophenolate mofetil versus cyclosporine (NEORAL)/sirolimus in renal transplantation. II. Survival, function, and protocol compliance at 1 year. *Transplantation*, 2004;**77**:252.

Ciancio G, Burke GW, Gaynor JJ, et al. A randomized long-term trial of tacrolimus and sirolimus versus tacrolimus and mycophenolate mofetil versus cyclosporine (NEORAL) and sirolimus in renal transplantation. I. Drug interactions and rejection at one year. *Transplantation* 2004; **77**: 244.

**Lorber-2005(B251)**

Lorber MI, Mulgaonkar S, Butt KMH, et al. Everolimus versus mycophenolate mofetil in the prevention of rejection in de Novo renal transplant recipients: A 3-year randomized, multicenter, phase III study. *Transplantation* 2005; **80**: 244.

Kaplan B, Tedesco-Silva H, Mendez R, Kahan B, VanBuren D, Boger R. North/South American, double-blind, parallel group study of the safety and efficacy of certican (rad) versus mycophenolate mofetil (mmf) in combination with neoral and corticosteroids [abstract]. *Am J Transplant,* 2001;**1(suppl 1)**:475.

**Vitko-2006(TERRA)**

Vitko S, Wlodarczyk Z, Kyllonen L, et al. Tacrolimus combined with two different dosages of sirolimus in kidney transplantation: Results of a multicenter study. *Am J Transplant* 2006; **6**: 531

**Vitko-2005(B201)**

Vitko S, Margreiter R, Weimar W, et al. Three-year efficacy and safety results from a study of everolimus versus mycophenolate mofetil in de novo renal transplant patients. *Am J Transplant* 2005; **5**: 2521.

Vítko S, Margreiter R, Weimar W, et al. Everolimus (Certican) 12-month safety and efficacy versus mycophenolate mofetil in de novo renal transplant recipients. *Transplantation*, 2004;78:1532.

Holmes M, Chilcott J, Walters S, Whitby S, Akehurst R. Economic evaluation of everolimus versus mycophenolate mofetil in combination with cyclosporine and prednisolone in de novo renal transplant recipients (Structured abstract). *Transplant Int*, 2004;17:182.

**Takahashi-2013**

Takahashi K, Uchida K, Yoshimura N, et al. Efficacy and safety of concentration-controlled everolimus with reduced-dose cyclosporine in Japanese de novo renal transplant patients: 12-month results. *Transplant Res* 2013; **2**: 14.

**Figure S1**. Egger’s test for biopsy-proven acute rejection.

**Figure S2**. Egger’s test for graft loss.

**Figure S3**. Egger’s test for new-onset diabetes mellitus.

**Figure S4**. Egger’s test for CMV infection.

| **Outcome** | **mTOR-I vs. MMF or Ec-MPS (Xie *et al*)** | | |  | **mTOR-I vs. MMF or AZA (Webster *et al*)** | | |
| --- | --- | --- | --- | --- | --- | --- | --- |
|  | **Trials (n)** | | **Relative risk (95% CI)^a^** |  | **Trials (n)** | | **Relative risk (95% CI)^a^** |
| Mortality (all cause) | 11 | 1.17 (0.89-1.53) | |  | 9 | 1.22 (0.78-1.90) | |
| Graft loss (all cause) | 11 | 1.20 (1.02-1.40) | |  | 10 | 1.13 (0.90-1.42) | |
| Death-censored graft loss | 9 | 1.31 (1.02-1.69) | |  | 9 | 1.01 (0.74-1.39) | |
| Biopsy-proven acute rejection | 11 | 0.99 (0.83-1.19) | |  | 9 | 0.82 (0.68-0.98) | |
| New-onset diabetes mellitus | 10 | 1.32 (1.07-1.62) | |  | 5 | 0.97 (0.68-1.39) | |
| CMV infection | 11 | 0.40 (0.27-0.59) | |  | 7 | 0.49 (0.37-0.65) | |
| Anti-lipid therapy | 8 | 1.35(1.18-1.54) | |  | 5 | 1.71(1.38-2.23) | |
| Thrombocytopenia | 3 | 1.97 (1.19-3.35) | |  | 4 | 1.95 (1.29-2.97) | |
| Leucopenia | 7 | 0.43 (0.29-0.64) | |  | 5 | 0.69 (0.44-1.10) | |
| Anemia | 6 | 1.21 (0.88-1.68) | |  | 4 | 0.94 (0.70-1.27) | |
| Impaired wound healing | 6 | 1.55 (0.97-2.47) | |  | 3 | 1.71 (0.88-3.32) | |
| Lymphocele | 7 | 1.80 (1.38-2.34) | |  | 4 | 3.07 (1.87-5.06) | |
| Malignancy | 8 | 0.64 (0.45-0.91) | |  | 8 | 0.83 (0.50-1.37) | |

Supplemental Table. Different result between our meta-analysis and the previous study

*Abbreviations*: Ec-MPS, enteric-coated mycophenolate sodium; MMF, mycophenloate mofetil; AZA, azathioprine.
